# Our child’s TBI: a rehabilitation engineer’s personal experience, technological approach, and lessons learned

**DOI:** 10.1186/s12984-021-00862-y

**Published:** 2021-04-07

**Authors:** James Sulzer, Lindsay S. Karfeld-Sulzer

**Affiliations:** 1 Department of Mechanical Engineering, The University of Texas at Austin, 204 E. Dean Keeton St. ETC 4.146D, Austin, TX 78712 USA; 2 TeVido BioDevices, Austin, TX USA

**Keywords:** Pediatric rehabilitation, Traumatic brain injury, Personal experiences, Technology-aided therapy, Assessment and assistance

## Abstract

I (JS) am currently a faculty member at The University of Texas at Austin in Mechanical Engineering. My primary research focus is rehabilitation engineering. In May 2020, a week before her fourth birthday, our daughter suffered a severe traumatic brain injury in the early days of the coronavirus pandemic. The purpose of this article is to describe the current state of pediatric neurorehabilitation from technologically-adept parents’ first-person perspectives in order to inform and motivate rehabilitation engineering researchers. We describe the medical and personal challenges faced during the aftermath of the accident, the technological approaches to her recovery that my wife (LKS) and I have examined, some of which may be considered beyond standard practice, and the lessons we have absorbed during this period regarding both the state of rehabilitation research and the clinical uptake of rehabilitation technologies. We introduce a set of questions for designers to consider as they create and evaluate new technologies for pediatric rehabilitation.

## Introduction

### Brief biography and introduction

Presently, I (JS)^1^ am faculty in Mechanical Engineering at The University of Texas at Austin, and my wife (LKS) is the Chief Technology Officer at TeVido BioDevices. My background is in rehabilitation engineering, primarily aimed at recovery after stroke, and my wife specializes in regenerative medicine. In this commentary, we will describe our experience with our daughter’s recent traumatic brain injury (TBI) starting with the facts of the case (“Facts of the case” Section), followed by a description of the medical and personal challenges (“Unmet Medical and Personal Challenges” Section), a summary of the different technologies used in the clinic (“Role of technology in clinical practice” Section), a list of the technologies we’ve applied beyond the clinic and based on this experience (“Technologies Explored” Section) in reference to the principles of rehabilitation devices that we have developed. Some of the descriptions may be somewhat jarring to a casual reader, and were indeed alarming to us, but we feel it is important to describe this experience as objectively as possible so it may be of use to the rehabilitation community. For other parents and caregivers, we hope this article provides a helpful perspective on incorporating technology into a therapy regimen.

### Facts of the case

In May 2020, our daughter, B,^2^ nearly aged four years, was struck on the head by a large falling tree branch while playing in the backyard with her older and younger brothers. She was knocked unconscious and her skull was fractured. At time of arrival at the emergency room at Dell Children’s Medical Center (DCMC) in Austin, TX, B had a blown right pupil, indicating brainstem dysfunction and the necessity to intervene immediately to avoid death. She received a craniectomy, a removal of a portion of the skull, to relieve the pressure on her brain.

B spent two weeks in a coma in the pediatric intensive care unit where she began to develop tone, first after a few days in her plantarflexors, and then after two weeks in her biceps. We conducted passive stretching three times a day for about 45 min to try and avoid her developing contractures, a shortening of the muscle fibers. Once her intracranial pressure stabilized and she could breathe without a ventilator and oxygen assistance, she was then transferred to physiatry for approximately 6 weeks at DCMC. B received a cranioplasty to replace her skull, a gastronomy tube (g-tube) for feeding/medications, and a ventriculoperitoneal (VP) shunt to drain cerebrospinal fluids. She engaged in therapeutic exercise similar to a typical inpatient schedule (3 h total of physical therapy (PT), occupational therapy (OT) and speech therapy (ST) daily). After those two months, B was classified to be in a murky cognitive area between a wakeful unresponsive and minimally conscious state. Once she was medically stable, we transferred her to inpatient care at Kennedy Krieger Institute (KKI) in Baltimore, MD for 9 weeks due to their specialization in disorders of consciousness in pediatric TBI [1]. There she received at least 3 h of PT, OT and ST daily, but also engaged in up to two additional hours daily of therapeutic recreation. Once her medication regimen was stabilized to promote her best chance of recovery at home, she was discharged home to Austin in September, about 4 months after the injury.

At home, B began outpatient therapy at DCMC for 7 h/week and simultaneously participated in a home therapy program for 9 h/week (ST, OT, and PT). We hired caregivers, mostly speech-language pathology (SLP) graduate students, to come over on evenings/weekends for 15 h/week where their main focus was therapeutic recreation. We enrolled her in vision therapy once every two weeks. We additionally carried out home exercises at approximately 10–15 h/week. A faculty colleague came once a week for an hour of PT. After 3 months at home, B’s therapists thought she was making strong gains so she was admitted back into inpatient therapy at DCMC for an intensive “boost” for two weeks (15 h/wk with the same ST, PT and OTs). We then directly followed her inpatient boost with 3 weeks of intensive outpatient therapy (20 h/wk with same ST, PT and OTs) at NAPA Center in Austin.^3^ We were approved for Medicaid waiver disability services over 8 months after the accident and are now eligible for home care including nursing and personal attendants.

Her current level of motor impairment at the time of this article (9 months post-injury) is severe. Unfortunately, we lack appropriate quantitative clinical assessment methods to succinctly describe her level of function. To informally describe these impairments, she can reach targets with her left leg and right arm when given sufficient time and within their somewhat limited ranges. She can produce limited power grasping with her right hand and some wrist supination. She has no ability to hit targets with her left arm/hand and within a small range of motion can hit targets with her right leg. She can take some self-initiated steps when fully supported. She can hold her head and trunk up for a short period of time. She can roll from front to back and back to front over both shoulders. She has a delayed and inconsistent swallow, so currently receives all food, water, and medications through her g-tube.

Cognitively, B tracks with her eyes, recognizes familiar faces, laughs at some jokes and even makes some of her own (nonverbally), enjoys watching kids’ shows, and follows commands within her abilities. She has a limited understanding of language. She vocalizes intentionally, but cannot say any words. She has cortical vision impairment, but the extent of this impairment is currently unknown. She remains severely cognitively impaired, although due to motor and speech impairments as well as being so young, it is difficult to assess the degree of her consciousness.

## Unmet medical and personal challenges (“she recovers what she recovers”^4^)

The purpose of this section is to outline some of the challenges we have encountered so far. Since this is written for an engineering audience, we wanted to provide a comprehensive picture of the problem to inspire some solutions. But we also wanted to share the personal challenges that are an integral part of pediatric rehab and should therefore be considered during solution design.

### Medical challenges

#### Motor and cognitive functional level

Perhaps the most visible challenges are B’s motor and cognitive impairments. She is fully dependent for all activities of daily living. In some ways it is similar to caring for a newborn, but substantially more complex. Communication is challenging. She can select between two choices (e.g., more/all done, stop/go, different toys), and displays emotions such as happiness, aversion, discomfort and pain. Often, she is upset for unknown reasons, but a soiled diaper, being left alone, bored of a TV program, uncomfortable position, pain, nausea or constipation are most likely reasons.

B does not understand that training will make her better so she needs the proper incentives to participate. She likes family pictures and videos, bubbles, breaking things (e.g. block towers), music, looking in the mirror, attention, and seeing people get hurt (minorly). However, she habituates quickly to these rewards, which need to constantly change (about every 5–10 min). Exercises that she seems to enjoy the first 2–3 times or even more soon elicit little to no active participation. Reduced endurance also likely plays a role.

#### Musculoskeletal issues

B developed severe osteoporosis in both distal femurs after 2.5 months post-injury. As a result, we need to manage and be acutely aware of loading on her legs, especially normal loads and twisting loads. She also developed heterotopic ossification in her distal radii, a condition where the bone fuses into the muscle. This likely is preventing her from fully extending her elbows, in addition to the contractures that have developed. Her right plantarflexors likely have contracture and she is undergoing serial casting to slowly stretch the muscle. It also appears that the skull is resorbing from the earlier cranioplasty and will require future plastic surgery.

#### Implants

B has two implants: a VP shunt and a g-tube. The VP shunt carries cerebrospinal fluid from her ventricles to her abdominal cavity to avoid excessive intracranial pressure. Shunts often fail over months and years, and it is difficult to know if a failure has occurred unless symptoms such as vomiting or a change in cognitive state appear; if so, this is an emergency. The g-tube is an implant in her abdomen that provides a port to her stomach for purposes of feeding and medicines. The g-tube can be pulled out fairly easily due to mishandling during transfers, gets clogged, and gets pulled out sometimes by our daughter. It is also an infection risk.

#### Clinical evidence

Initially we were adamant that B receive evidence-based treatment, especially given knowledge that the field has been dominated by eminence-based medicine, that is, the opinions of respected clinicians, for years. Unfortunately, there are very few clinical trials in pediatric TBI in comparison to cerebral palsy (CP), stroke, or even adult TBI. We were not made aware of any research studies that we could participate in during our stays in the hospitals. Despite our knowledge of the advances that come from research, we would most likely have declined participation in these studies due to the overwhelming stress of our situation. If it was a late stage clinical trial with more knowledge and likelihood of success, we still would have feared receiving the placebo. While clinicians rely on relatively conservative treatments based on stroke and CP literature, it is certainly possible that for many of these treatments, individuals with pediatric TBI may not follow suit. This leaves us with very little factual information to decide the best course of action and vulnerable to individuals claiming efficacy of their treatments without evidence.

There is also a lack of knowledge of how neuroimaging measures correlate to recovery. Perfusion imaging was used to identify permanently damaged areas of her brain, and I additionally asked for white matter tract imaging during her inpatient stay. However, this request was refused because it would not affect her treatment, and along similar lines it is unclear how information from perfusion imaging played a role in her treatment. Thus, current clinical practice treats the brain as a black box and “she recovers what she recovers”. Ironically, this is usually what I lead my grant proposals with to motivate why we need to better understand how the brain recovers from injury. I was hoping that this experience would provide some new insight in that regard, but instead it has only reinforced the need for more quantitative metrics for tracking impairment and recovery.

#### Alternative treatments

Along with the lack of clinical evidence for standard treatments, there are a host of alternative treatments that have scant clinical evidence but need to be explored. Among those that we researched include hippotherapy (i.e. horseback riding), infrared laser therapy, essential oils, stem cells, hyperbaric oxygen therapy (HBOT), and various alternative physical therapy approaches. Some of them, including HBOT and stem cell therapy, have clinical evidence for other indications, but little to no data for TBI and/or in the pediatric population. Stem cell treatment has few practitioners and there are fewer clinical trials that are ongoing. HBOT is popular with parents, but not recommended according to a recent review in CP literature [2]. Many parents of children with TBI and other brain injuries report success with stem cells, HBOT, and other therapies, but there is no control and the potential for a placebo effect. This situation creates a conflict between how we were trained to evaluate evidence as scientists and wanting to provide whatever therapy could potentially help B. For instance, B could be one of the responders to treatment even if the average patient may not benefit. There could also be nuances in these clinical trials that could indicate B would benefit but could not be gleaned from a high-level review and we do not have details of the literature that could help us make a more informed decision. Additionally, the lack of data to support a therapy in this indication does not mean that it is not effective, but it requires research. Speaking with knowledgeable colleagues on the subject is helpful, but there are also conflicting opinions. Moreover, these alternative treatments require considerable time and money and are not covered by insurance.

### Personal challenges

#### Patient time

Aside from an unknown window of neuroplasticity, B’s severely impaired functional state implies a host of training exercises are needed. Vocalization, stretching, swallowing, sitting, standing, walking, grasping, rolling, vision training, decision making, are some examples, and this training occurs at home and in the clinic. When factoring in feeding times (4 per day at about 1.5 h per feed), napping (1 h), and transportation to the clinic (about 20–30 min each way, occasionally multiple trips), there is not enough time in the day to accomplish all the exercises required. We also need to consider her tolerance for all of the training.

#### Parental time

Simply put, the caregiver burden is crushing. As parents we need to take on the additional roles of advocate, administrator, nurse, and therapist often simultaneously and requiring both parents, taking attention away from our two other children. Transportation and attendance for her at school, medical appointments including multiple specialties, orthotics, PT, OT and ST is time consuming. Additional tasks including researching alternative therapies, researching and organizing caregiver, medical, educational and nursing plans, navigating labyrinthine Medicaid and Medicaid waivers with little knowledge, refilling prescriptions and unexpected ER visits are many examples of time requirements. We took a leave of absence for an extended period of time from our jobs or were not working. Community and family support are critical, but limited in reducing this time burden. This issue is especially pronounced since we have no family in the area, but we are deeply thankful to have a supportive community locally and friends and family all over the world that have supported us in a multitude of ways. The time strain affects regular exercise, our own medical appointments, housework, sleep, relaxation, careers, and time to spend with our other children and our marriage.

#### Adjusting to a new lifestyle

After returning home to Austin, we were thrown into a new lifestyle. Up until three months ago B had to be turned in bed every four hours including in the middle of the night. She currently receives about 175 doses of medication a week at six different times of day. We needed to acquire adaptive equipment as soon as possible, despite insurance taking months to supply an adaptive stroller and stander. We were able to get by through a local equipment sharing program (CPath) where we could find a bath chair, wheelchair and stander that were good enough in the short term. Adjusting our lives to the coronavirus pandemic was certainly challenging, and then this adjustment was bewildering. Although we had basic training at the hospital, much of what we learned was from trial and error, which can produce some scary moments with B in such a fragile state. We needed to learn numerous tasks that were new to us, e.g. administering medications, g-tube care, managing her health care schedule, coordinating between insurance and medical providers, etc. Typical activities also became more challenging (e.g. getting dressed, brushing teeth, riding in the car). Fortunately, our house and car didn’t require modifications due to how they were already configured and her small size since she is so young.

#### Insurance and expenses

There is a substantial cost of care even with insurance. Expenses include splints, outpatient therapy, home therapy, stroller, stander, medicines, and specialist appointments. We provide a co-pay for these items through primary insurance, but even with a co-pay adaptive equipment can be expensive (e.g., our stroller co-pay was $1200, our stander was $800). Our primary insurance does not cover items that we feel are essential such as car seats, bath chairs, and vision therapy. The treatment costs are exacerbated by lost wages as well as additional costs for our other two children. Primary insurance will only cover up to 9 h of outpatient therapy a week, substantially lower than what she needs. We were able to obtain an additional 9 h per week of home therapy through a different benefit category, but we were told this is a rarity, which was surprising given its ability to aid recovery. The desire to provide the very best treatment occasionally clashes with out-of-network options such as intensive outpatient programs. Understanding insurance reimbursement is a very nebulous process, sometimes even to the insurance company employees themselves, which has caused some issues. Receiving public assistance for a disability is complicated and potentially a long wait, making it a difficult process to navigate alone. Eligibility for Medicaid, Supplemental Social Security, and Medicaid Buy-In is dependent on the income of the entire family, which must be below a certain threshold. This income limit is at maximum only about 1.5 times the federal poverty limit, so families with higher incomes could still have detrimental impacts of medical bills, caregivers, and lost wages. Medicaid waivers are available for people with disabilities and only based on the individual’s income, but may have years-long waitlists,^5^ dependent on the state. We recently received Medicaid approval which will help reduce costs and provide caregiver support. We are hopeful that this will ease the burden.

#### Emotional trauma

Everyone in the family was traumatized by the accident. Acceptance of such a rare accident radically changing all of our lives remains difficult, but was helped by acknowledging that B as we knew her is gone. Thus, to a large degree, it feels like losing a child. However, this is an ambiguous loss since we don’t know to what extent she will recover, complicating the grieving process. Adding to this trauma is the uncertainty of whether our family will be able to participate in the activities we envisioned, e.g. vacations, hiking, sibling play, or how it will affect career choices, ability to move to a new location, or other life decisions. While initially it was frustrating that clinicians could not offer a prognosis, later we realized that such a prognosis has limited utility because it is only a prediction and will not affect how we treat her. Although we try to remain hopeful, it is very challenging given the slow pace of improvement and general anxiety. The emotional trauma affects our response to additional issues that arise (e.g., violent vomiting, accidental g-tube removal, new surgeries) as well as the care for our other children. Certain stimuli can trigger memories of times prior to the accident: a dress, her favorite foods, another child, annual traditions and events, etc., all causing emotional distress. There is also a strong feeling of helplessness throughout her experience, perhaps exacerbated by the proximity of my professional expertise to her injury. Activities such as searching out advice from colleagues, developing rehab devices, communicating my experience in coursework and writing this article have been therapeutic.

#### Effects on siblings

As noted above, both of our sons witnessed the accident and the following tumult. While it is unclear how cognizant our younger son was, our older son was quite aware something was terribly wrong. Explaining why he could not see his sister for weeks and the state she was in was more difficult for us than for him, but he still missed his best friend and was not fully aware of how impaired she was. After returning home, our oldest did his best to care for B and spend time with her, seemingly very mature for his age. However, compounded by the social isolation of the pandemic, there were many instances of him and his little brother acting out in situations that would seem unwarranted. He was aware that his sister and little brother both need a great deal of attention, but that still does not mean he accepted not getting enough attention from his parents. He talks to counselors to help him communicate his feelings since this does not come naturally to someone his age. Despite occasional tantrums we are amazed at how well he has fared in this situation. Although separation anxiety is somewhat appropriate for someone our younger son’s age, his difficulty leaving us seemed particularly heightened.

In summary, these challenges represent a large portion of what makes this experience so difficult. What should also be mentioned is the interactive nature of these challenges. For example, reduced sleep and increased stress has caused mistakes with her medications or splinting which have negatively impacted B. We can only speculate as to how difficult these challenges must be to parents of lesser means and/or support structure. It would have been helpful to have a smoother, expedited path to Medicaid and a faster, more transparent decision-making process from our primary insurer. While we received basic care training before discharging from our inpatient stay, a nurse coming to our home to show us best practices on how to take care of her would have eased the transition. We were fortunate to have an experienced OT serve as a coordinator on a *pro bono* basis that helped us communicate with adaptive equipment, home therapy and insurance. While we used our connections with the scientific community to investigate alternative treatments, we would have appreciated a resource curated by clinical and scientific experts, possibly even an interactive resource, that could help us determine whether a certain treatment might be beneficial in B’s case.

## Role of technology in clinical practice (“meet her where she’s at”)

The purpose of this section is to detail the rehabilitation technology that B was exposed to in the clinic during her therapy experiences thus far.

### Electronics

The most commonly used electronics were iPads, eye gaze tracking, and switches that start/stop videos or activate toys (cause-effect). iPads were most often used to play movies or songs to provide motivation. Sometimes these movies were played on a computer with a button switch that could pause or play as an incentive. iPads also had nice cause-and-effect games (e.g., “Talking Carl” by Tayasui.com) that B found funny. One idea we felt would be helpful is a suite of iPad apps centered on therapy rather than adapting existing games. Especially during the pandemic, iPads were used for teletherapy; for example, music therapy with Nationwide Children’s.

Motorized toys such as a cow were used with a wired remote button for stop/go commands. Having a 3D object and not staring at a screen was sometimes more appealing for B. We have learned that to operate these toys with a large button switch appropriate for our daughter and other children like her, many of these types of toys need to be adapted to a switch aftermarket, which can be costly for some clinics. Clinics could likely use a program that adapts such toys for them, perhaps of interest to mechatronics students.

Eye gaze tracking technology is our current strategy for communication. She is currently learning standardized templates of words using UNITY (PRC Accent 1400) which shows a grid of graphics and the user learns to identify the graphic with the meaning of the word based on the motor memory of the location and audio feedback. She plays games on the system such as looking at a person’s face, with a successful hit rewarded with a virtual pie splat. There is a lot of effort on behalf of the alternative augmentative communication (AAC) specialist to teach B the meaning of the templates and it is a slow process. We have struggled with calibration and have often needed the AAC specialist to perform one on herself, perhaps reducing the performance of the eye tracking. We found having a trace of her current visual gaze position on the screen was critical to evaluating her performance, yet some software lacked that capability.

### Peripheral neurostimulation

Electrical stimulation is on the fringe of what we would consider conventional therapy, but therapists seem to be well aware of it. AC stimulation in the form of transcutaneous electrical nerve stimulation (TENS) and neuromuscular electrical stimulation (NMES) is also used in the clinical setting. These techniques use the same hardware, stimulators operating on a 9-V battery that are often available at a relatively low cost. We briefly used TENS on B’s quadriceps to reduce spasticity and recently used NMES under her chin to encourage swallowing. It is difficult to tell if TENS is effective, however there did appear to be some immediate effects from NMES under her chin.

Vibration therapy has some evidence (e.g. [3]) but is also considered fringe. We used a vibration plate in the clinic on both her upper and lower limbs to reduce tone, provide proprioceptive input, and build strength. Vibration was set to about 20 Hz. B tolerated it well, although that does not seem to be the case with all children we observed.

### Devices to support posture and gait

Positioning devices are critical to her recovery. Standers that worked best have the most adjustments, which has the relatively minor setback of extra bulk and weight. We currently have the EasyStand Zing stander. Often even with the adjustments, more modifications are needed such as extra padding to keep her knee in a neutral position and padding on the footplate when she is on her bare feet. Standers are very useful because they provide a benefit such as weight bearing while enabling other exercises or simply just watching TV.

A good stroller also makes a huge difference. Having a comfortable stroller with supports and adjustments in the right places is critical for everyday living, sitting at the dinner table, participating in vision/speech therapy, or simply maintaining an upright posture to prevent orthopedic spine issues. We have the Convaid Trekker, which has two bases, one for outdoor use and one more mobile and with height adjustment for indoor use. While effective, the stroller takes up the entire trunk in our minivan and weighs about 60 lbs.

For B’s excessive plantarflexion tone, she has undergone several bouts of serial casting. In this procedure, the ankle is put into a cast with a slight dorsiflexion force for three days to a week, followed by another cast ideally at a higher dorsiflexion angle. This continuous, light force is intended to prevent or assuage her plantarflexor contracture. We timed it with Botox injections and have been able to improve dorsiflexion almost to neutral position to enable better gait training. For upper limb tone, we have been using splints, most often at night, to prevent contractures. She has a wrist splint and elbow splint. In the pediatric intensive care unit, we did not perform casting, but instead stretched her muscles by hand. It is very difficult to evaluate how effective this stretching was, especially since B may have developed a contracture in her right plantarflexors. There could be an opportunity there for automatized dynamic stretching devices applied at a low force for a longer period of time.

In the clinic, B has engaged in locomotor therapy which was often enabled using a walker or bike. Various unmotorized, wheeled walkers have been used, including ones that support her weight under the arms, her trunk, and others at the pelvis. Most of the devices provided full body weight support (BWS) because that is what she needed, but some were capable of partial BWS. Along similar lines, she also received bike training with a modified tricycle that supported her at her trunk and allowed her to transport herself. While still at limited mobility, she seemed somewhat motivated to use these devices.

I have been around robotic rehabilitation devices my whole career, so I was heartened to see them in the clinic. There were two devices that we observed, a planar arm robot and a pediatric gait exoskeleton. B was too impaired at the time to use the arm robot and since she needs to practice active leg motions and foot placement, the exoskeleton did not seem like a good fit. We should note that after over 9 months in several pediatric clinics, we have not yet seen any robot being used. While robots have been acquired by many institutions, it appears that they are not yet the centerpieces initially envisioned. Overall, we found the use of technology to be fairly limited during rehabilitation. The therapist expression, “low-tech is often the best tech” holds true because low-tech is reliable, rarely breaks, is cheap, can be easily fixed/replaced, often serves multiple purposes, and can be used effectively by a creative therapist. We often describe robots as tools for therapists, but therapists’ tools are a bench, mat, ball, roll, and table. I myself would be skeptical if someone told me to use a robot in my mechanical workshop instead of my screwdriver or hammer which provide sensory feedback. The experience has encouraged me to reevaluate what innovations in rehabilitation should look like, as detailed in the next section.

## Technologies explored (“leave no stone unturned” and “there is no playbook”)

The feeling of helplessness and rollercoaster of emotions is often temporarily assuaged with new treatments and devices. Gaining a sense of agency over her recovery is therapeutic, but development and testing of new techniques can waste time otherwise spent on her immediate needs. Some themes of why some techniques stick and others do not have emerged to us. In this section we compile these principles as a list of questions that we think rehabilitation engineers should ask themselves during the brainstorming process.

One of the inspirations has been the hours of observing B’s recovery and the therapist interactions. Despite much of my career spent working aside therapists, I did not develop a sufficient appreciation for what therapists are capable of and the physical and mental demands of the job. Effective therapy requires resourcefulness, creativity, intuition, problem-solving, ability to motivate, energy, a positive attitude, cooperation/teamwork, communication, personal connection and patience. It also can require dexterity, flexibility, and strength. From my perspective it is abundantly clear that despite the fears of some therapists, robotics/technology cannot replace them in the foreseeable future. That being said, therapists vary in attitudes towards technology, and careful consideration needs to be made towards how the technology is introduced. For instance, just placing a new device in a clinic is not enough; it needs to be demonstrated how the device will substantially help the patient and preferably make the therapist’s job easier.

**Fig. 1:**
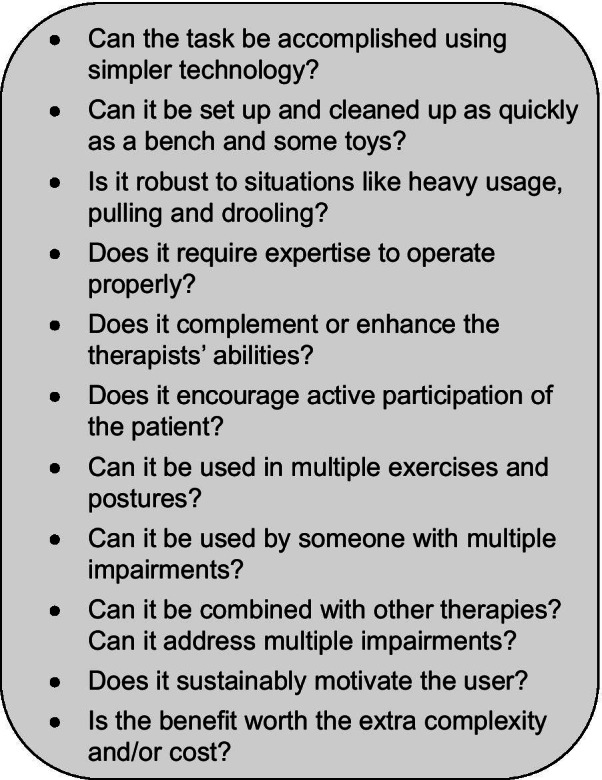
Questions for rehabilitation technology developers

Based on our experience, we propose that the following set of questions are particularly important to address during the design and evaluation process (Fig. 1). For example, we found the more complex the technology, the more often something went wrong, and then the more the device went unused. Considering a very busy schedule, anything with a setup time longer than a few minutes required planning and hindered use. However, if the device could be combined with other devices/therapies simultaneously, this saves time and motivates us more to use it. Furthermore, if the device could address multiple impairments, e.g. reaching and vision, simultaneously, this also saves time. Being flexible enough to be used in multiple postures and for different exercises promotes usage. Motivating B for a sustained period is a constant challenge, thus help from a device to support motivation is helpful. The following describes some of the technologies we explored that are often not part of the standard-of-care. Some are commercially available, and some have been developed by us or colleagues. In this section we evaluate these approaches qualitatively and then summarize their performance with respect to the questions in Fig. 1.

### Lower limb approaches

#### Home BWS system

**Fig. 2:**
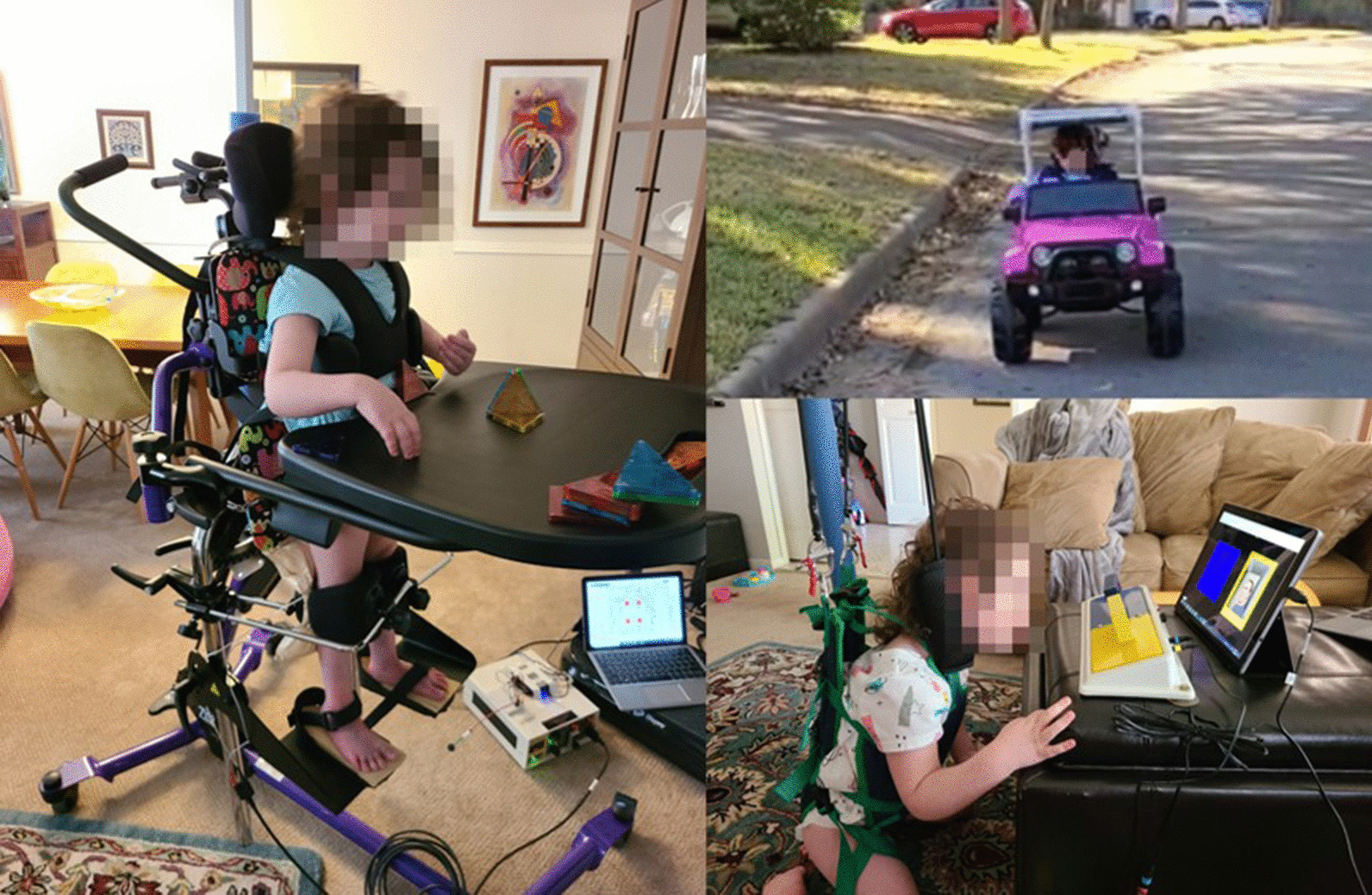
(Left) B in stander with DC electrical stimulation and pressure sensing while engaging with one of her favorite toys, Magnatiles. (Top right) B in a modified car where she uses her head to push a button that accelerates the car. Car is poorly steered by me. (Bottom right) B performing decision making and upper limb motion with a custom two-button device while wearing a BWS system to help maintain a kneeling posture

BWS systems are used to provide unweighting to patients to enable gait therapy earlier in the recovery process. We acquired a low-cost BWS system (PUMA from Enliten, LLC) that supports weight with bungees and allows translation in two degrees-of-freedom within a 9′ × 9′ area. We used it for sit-to-stand training and gait training, but also used it for exercises where we wanted extra trunk support but did not have an extra pair of hands (Fig. 2). We also put the fasteners on the back of the harness and used it for crawling training. We found this system to be well-engineered, multipurpose and relatively easy to set up in under 5 min, although the area of usage was restrictive.

#### Mobility harness

Gait training is usually carried out with the therapist facing the patient, but with a mobility harness, the patient is harnessed to the parent/therapist with the patient’s back against the parent/therapist’s front, akin to a tandem skydive. We used the Upsee Mobility Harness by Firefly which allows one of us to walk along with B harnessed to our waists. The system uses connected sandals that fit each of our feet to a single rigid platform. Setup time was a bit longer than the BWS system at about 5–10 min. We both enjoyed using this harness because it allowed B to walk anywhere and with our assistance, she could walk further and explore. We were able to use small haptic cues (i.e. ankle plantarflexion) to help initiate steps and used our knees to correct her pelvis. In contrast to a pediatric exoskeleton that restricts motion to the sagittal plane, controls foot placement and does not provide direct force feedback to the therapist, the mobility harness seemed to better promote active participation and maintained force feedback. However, the force feedback was not always detectable due to her small forces and her knee extension was not visible to inform us if she was weight bearing properly. More importantly, being tied to a fragile smaller person created a potential safety hazard, especially if falling or stepping too widely, so extra care was spent to take careful steps.

#### Vibration

Whole body vibration can improve spasticity, balance, strength and possibly bone mineral density [3]. In addition to vibration therapy at the clinic, we purchased a vibration plate at home (LifePro waver) that ranges from about 8 Hz to 12 Hz. Based on earlier work [4], we set it at about 10 Hz for 2 min duration. We used focal vibration of the triceps during stretching for three 30 s bouts using a basic vibration massage tool available at drug stores. Both of these were very quick to set up (1–2 min) and tolerated well.

#### Office bike

We purchased an office bike (Sanxia) that B used while in a bean bag chair to encourage reciprocal voluntary lower limb activation. Even without voluntary activation, our hope was that such reciprocal passive motion would improve motility and mobility in her legs. We secured her feet to the pedals with tape and encouraged active participation by playing B’s favorite music only if she was moving. Even still, motivation was the most difficult challenge to participation. Future plans may include attaching it to her stroller or converting to a functional electrical stimulation (FES) bike using the AC stimulation [5]. Another option is a custom tricycle (e.g. Amtryke from AMBUCS organization) that could additionally enable mobility.

#### DC peripheral nerve stimulation

We attempted to offset plantarflexion tone by exciting ankle dorsiflexors and reciprocally inhibiting ankle plantarflexors via DC electrical stimulation of the common peroneal nerve. We coupled the stimulation with use of the stander in order to induce dorsiflexion through muscle activity as well as through external forces provided by the pedal of the stander. We employed stimulation in the form of 5 pulses of 100 ms width at 20 Hz separated by about 6 s just above the motor threshold (~ 6 mA). We used a Digitimer DS7A with a National Instruments data acquisition system (NIDAQ) and custom Matlab code (Fig. 2). Setup time was about 5–10 min because finding B’s small common peroneal nerve was difficult. It was hard to visualize whether the stimulation was inducing desired changes, so we developed a force sensing foot plate to quantify it.

#### Pressure sensing

We designed the custom force sensing foot plate to detect distribution of pressure between the ball of her foot and the heel while in the stander to assess if there was improved weight distribution. We sandwiched Flexiforce sensors (Tekscan) between two layers of thin silicone topped by a rigid board to distribute weight. We connected the sensors to the NIDAQ and then integrated to the same Matlab code running the DC stimulation, presenting the data graphically using a colorbar. The goal was to be able to quickly assess force distribution anterior/posteriorly on her most affected right foot. However, we also wanted to assess weight distribution between B’s left/right sides during standing to make sure her osteoporotic femurs were being evenly loaded. Setup time was less than 3 min. We were able to use this setup while running her medications and feed. Since these tasks don’t require her attention, we have further simultaneously engaged a reaching and grasping task while performing vision therapy with an eye patch. The multitasking saved much needed time.

### Upper limb approaches

#### Markerless motion capture

As part of a class project for a student, we tracked the recovery of B’s head movement ability using a stimulus. The stimulus was the motion across her field of view of her favorite videos. We put a pattern on her forehead printed on paper and secured with a headband and recorded with a webcam. Setup was simple. However, while the head turned from side-to-side, the pattern was often lost by the analysis software. We had a more difficult time with the same issue using markerless facial recognition software.

#### Two-button interface

B is learning to make choices, often between two objects. It is difficult to know if she moves a direction due to motor impairment or some other non-decision related variable. To engage her and teach her to make decisions, we developed a two-button interface. Using a commercially available two-button switch (Rocking Say It Play It by Enabling Devices), we made custom Matlab software that presented two choices on a screen behind the buttons (Fig. 2). Each choice had a background color matching the buttons (i.e. yellow/blue). One of the choices on the screen had a family picture overlaid while the other was left blank. When the correct button was pressed, a voice recording of the family member was played. After three months, this remains one of B’s favorite toys, but it is only effective for about 15 min each time we use it. It takes 5 min to set up and revolves around a delicate laptop so there are issues with setup time and robustness.

#### Modified ride-on car

We would love B to independently move and explore where she wants to go. As part of a senior capstone project, students modified a motorized toy ride-on car to incorporate a large button fixed to the roof of the car that moved the car at a single speed based on plans from the Go Baby Go program (Fig. 2). The goal was to encourage her to control her head position. We controlled the steering and emergency stops with a remote control. When she was motivated to use it, she found it very enjoyable. However, it was difficult for us to tell if she was moving due to impaired head motion accidentally hitting the button or if the movement was voluntary.

#### EMG biofeedback

B has many difficulties with coordinating muscle activity. One of my graduate students developed a custom electromyographic (EMG) biofeedback system based on Arduino that adjusted the volume of her favorite songs based on EMG activity of a single muscle or the difference between two muscles (i.e. agonist–antagonist pair). We used low-cost EMG sensors by Myoware. The goal was to encourage muscle activation in certain areas (e.g., neck) or discourage tonic activation (i.e., activate hamstrings, deactivate quadriceps). B was not cognitively ready for this type of technology and the EMG signal was too noisy, especially from her small muscles. Setup time was very long at about 10–15 min and it took an expert to use properly.

#### Virtual reality games

In order to gamify her arm motion, markerless motion capture seems to have the most promise because it adds no extra weight and has a quick setup. We adapted a game developed by a colleague using the Leap, an infrared camera that detects hand configuration and position. The game involves hitting asteroids in a virtual environment. Setup was simple; however, we were challenged by the camera losing sight of B’s hand due its small size and her difficulty in developing agency over the virtual hand.

**Fig. 3:**
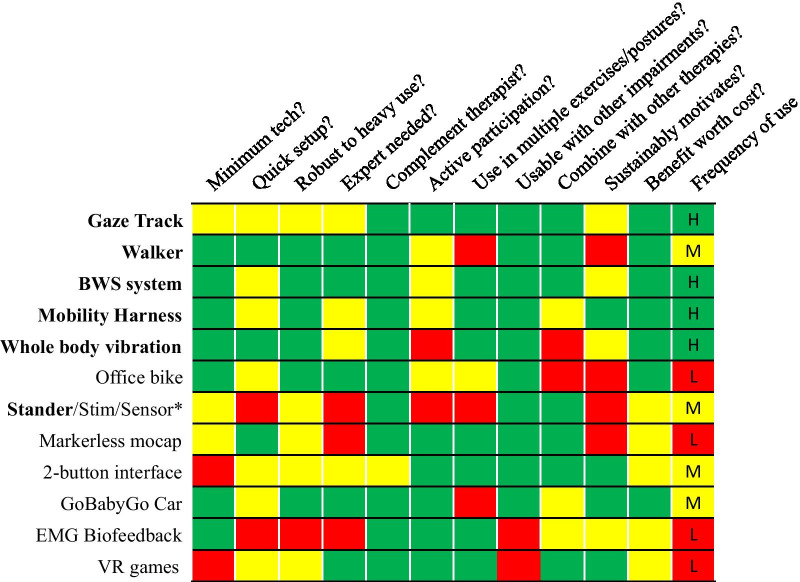
Summary of usability evaluation of selected therapy technologies used at home. Bold font refers to commercially available therapy devices. Other devices have been modified from their commercial version or constructed from parts. Green represents, “likely meets criterion”, yellow represents, “could possibly meet criterion with further development” or “occasionally meets criterion”, and red represents, “unlikely to meet criterion”. “Frequency of use” refers to our use at home with no scheduled therapy. “L” refers to low (rarely), “M” to medium (at least every 1–2 weeks) and “H” to high (at least every 2–3 days). *Stander is used every day, but not always with electrical stimulation and pressure sensors

We have listed some selected commercial approaches along with ones we developed and evaluated them against the questions in Fig. 1 according to our experience (Fig. 3). These evaluations are meant to encourage thought on how these technologies fare according to our basic principles but are not a judgement on the therapeutic success of the approaches which would take far longer to evaluate. This is the first step in a long road of investigation of technologies. Some future possibilities include use of force/motion sensors for games (e.g. Fitmi from Flint Rehab), a wearable vibration shirt, easy to apply inertial measurement units [6], transcutaneous spinal cord stimulation [7], and therapy toys that encourage range-of-motion, saccades, wrist supination, pointing, etc. In the future, we will continue to interact with the clinical and rehabilitation engineering community to find the most effective approaches.

## Conclusions

The challenges in dealing with a young daughter with a severe TBI are too difficult to entirely communicate within a commentary, but we hope to provide an initial grasp of what it entails. The ripple effects on the family and its entire social network are tangible. Unfortunately, there is a dearth of clinical evidence that shows clinicians and parents what treatments and strategies are most beneficial. Going forward, we hope researchers can collaborate more often across centers to develop the evidence so sorely needed in this field. Despite this lack of knowledge, we are using basic principles of neurotherapy and employing different technological approaches to facilitate them. We hope to report at some time in the future on the effectiveness of these strategies, how they have been integrated into our daily regimen, how our approaches have changed, and the substantial recovery of B.

Many of our previously held beliefs have been reinforced by this experience such as our need to understand how the brain recovers from injury, yet our attitudes towards technology, specifically in therapy devices, have become more critical. Most of the new ideas I have for devices for B are often abandoned because they do not hold up to the principles in Fig. 1. Developing useful rehabilitation technology is challenging and takes many iterations. In academia, I have found that we often overlook qualities of robustness, expense, simplicity and setup time in order to push the limits of technology. However, this immersive experience has helped us understand the need for such innovations in usability. Perhaps in the future, rehabilitation engineers in training could pair with a family and assist with caretaking and therapy duties for a period of time to gain a similar perspective. This would help develop a holistic appreciation of the challenges families and clinicians face and how devices would fit in with the daily regimen. As more advanced technology becomes clinically relevant, such as currently available gaze tracking, therapists will need to be trained not only on how to use it, but also in some of the engineering mindset, language and concepts. In addition to these considerations, we hope this article helps illustrate the challenges that the patient and patient’s family face and that these challenges are integrated into the design process.

## Data Availability

Not Applicable.

## Abbreviations

TBI: Traumatic brain injury
OT: Occupational therapy
PT: Physical therapy
ST: Speech therapy
SLP: Speech language pathology
DCMC: Dell Children’s Medical Center
KKI: Kennedy Krieger Institute
IMU: Inertial measurement unit
EMG: Electromyography
BWS: Body weight supported
CP: Cerebral palsy
AAC: Alternative augmentative communication
TENS: Transcutaneous electrical nerve stimulation
NMES: Neuromuscular electrical stimulation
HBOT: Hyperbaric oxygen therapy

## Footnotes

1 As a note, this commentary is written in the first person: “I” refers to one author (JS) whereas “we” refers to both authors (JS and LKS). We switch between the two pronouns as the situation requires; we realize this is a bit unorthodox.

2 This is a pseudonym.

3 B was treated by a small army of therapists since her injury. I often asked the therapists whether they follow any specific philosophy, e.g. Bobath, NDT, etc. No one I spoke to fully ascribed to any school of thought. The only consistent philosophy was that encouraging active participation was most important.

4 We were exposed to a whole new set of clichés during this experience. Each section has a corresponding cliché that will help you fit in at any rehab clinician cocktail party.

5 To illustrate the point, some waitlists are up to 15 years long.
